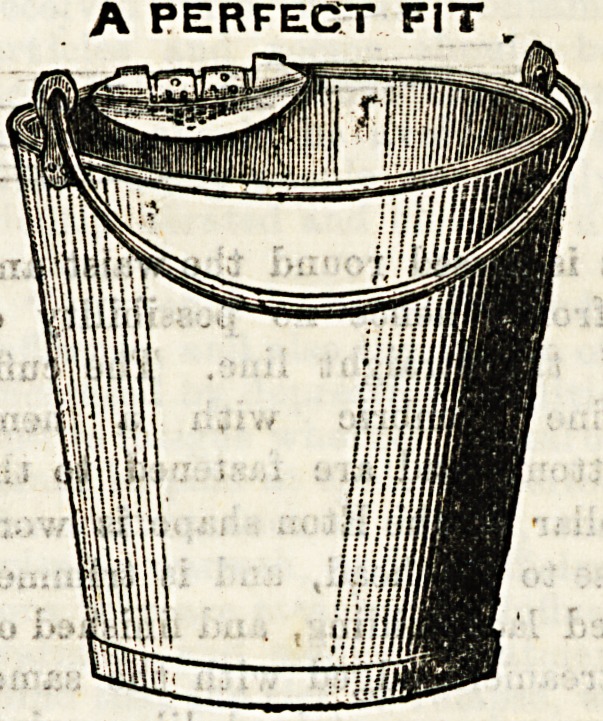# The Hospital Nursing Supplement

**Published:** 1895-03-16

**Authors:** 


### "The Hospital" Nursing Mirror.

Being the Extra Nursing Supplement of "The Hospital" Newspaper.

[Contributions for this Supplement should be addressed to the Editor, The Hospital, 428, Strand, London, W.C., and should have the word "Nursing" plainly written in left-hand top corner of the envelope.]

### News from the Nursing World.

### ROYAL VISITORS.

A visit was paid to the Chelsea Hospital for Women on Monday last by H.R.H. the Duchess of York, who distributed flowers to the patients, and expressed interest and pleasure in everything shown to her. H.R.H. the Duke of Cambridge went over the Civil Hospital at Malta last week; and the Empress Frederick has promised to visit the Cambridge Hospital at Aldershot on the 15th inst., when she will be the guest of their Royal Highnesses the Duke and Duchess of Connaught at Government House.

### PRINCESS CHRISTIAN AT OXFORD.

Dr. Liddell presided at the influential meeting held in the Sheldonian Theatre, Oxford, on the 8th inst., to discuss the memorial to Sir Henry Acland. In the course of the proceedings a letter was read in which Sir Henry Acland stated, "My preference, on public grounds, would be to increase and make permanent the Memorial Home for Nurses." It was therefore resolved unanimously, "That the testimonial should take the form of a provision for the increased usefulness of the Sarah Acland Home for Nurses, and without prejudice to any proposal for a personal memorial to Sir Henry Acland at the Museum." Princess Christian spoke of the value of trained district nurses, and of the esteem in which Sir Henry Acland's name and work were held. Her Royal Highness was greeted with much enthusiasm, and her short speech was received with great attention and interest.

### EAST LONDON NURSING SOCIETY.

An urgent appeal for funds is made in the last Annual Report of the East London Nursing Society. New subscribers are needed to replace those who have died, and it is stated that only by liberal support can the Society carry on its present scale of work. The expenses have considerably exceeded the income during the last twelve months, although the year began with a satisfactory balance in hand, and it is to be hoped that the annual meeting, held on the 12th inst., at the Mansion House, will arouse fresh interest and secure increased donations to the Society.

### LEWISHAM INFIRMARY.

In another column will be found two letters addressed by the Assistant Matrons to the chairman of the Lewisham Infirmary Committee. These apologies were not considered by some of the committee sufficiently humble, but no action was taken in the matter in consequence of the new feature introduced into the discussion by Dr. Pringle. He announced that certain letters now in his possession might affect the whole action with regard to the suspension of the Matron and the Assistant Matrons. In consequence of this important statement the Guardians referred the whole question back to the committee. Doubtless their report on it will be anticipated with much interest by our readers.

### ROYAL NATIONAL PENSION FUND.

The report of this Fund which appears in this week's issue shows it to be in a condition of prosperity such as seems to need but little comment. The facts and the figures speak for themselves, and the increased number of policy-holders gives complete evidence of a growing desire on the part of nurses to provide for a future "rainy day" when ill-health or advancing age may incapacitate them from work. Each year the benefits of this Fund have been more widely acknowledged, whilst its assured position lifts it above the reach of those petty and ignorant criticisms which have from time to time assailed it. The liberality with which numerous hospitals assist their nurses to become policy-holders forms a most substantial encouragement of thrift. Grateful indeed are the acknowledgments made by nurses for the Sick Pay received during enforced weeks of idleness when pecuniary anxiety would have added to bodily sufferings but for the existence of the Royal National Pension Fund for Nurses.—Several policy-holders, who have from various causes been obliged to withdraw, have shown their appreciation of the Fund by rejoining it at the earliest available moment. Any one fortunate enough to be a member should certainly hesitate before relinquishing its benefits.

### WAKE UP! EASTBOURNE.

We were much struck on a recent visit to the Midlands at the lively interest taken in the hospitals and their inmates by the wealthier residents. A fair proportion of the flowers grown in greenhouses throughout the winter are there sent to the hospital wards as a tribute of sympathy from the wealthy to the sick. Eastbourne is one of the most delightful of watering-places, and none in our islands surpass it in health-giving properties at this season. It contains very many beautiful residences, and the luxurious hotels and lodging houses have an abundance of beautiful flowers, but the sick in the hospital at Eastbourne which has, and is, restoring to health probably more influenza victims than any other seaside resort, are without one single bunch of violets sent by the wealthy from their abundance as a message of sympathy. Such forgetfulness is sad indeed. We venture to hope, however, that from this date forward the sick in the wards of the Princess Alice Memorial Hospital at Eastbourne will be cheered by an abundance of beautiful flowers from those who have more than enough, and who would be blessed in the gift with the receivers. The very wealth and beauty and health giving properties of Eastbourne cry aloud to its inhabitants and visitors to be up and doing in this matter, or the opportunity may escape them. Surely the gratitude of many a convalescent will thus find expression in simple but practical sympathy with the poor who are sick and in hospital. "I was sick and ye visited me."

### TRAINING AT THE LONDON HOSPITAL.

To provide preliminary training for pupil probationers is the object for which the committee of the London Hospital have recently taken Tredegar House. Whilst believing that sick nursing cannot possibly be properly taught without patients, Miss Lückes (matron of the hospital) considers that certain probationers' duties could be learnt before entering the wards. Possibly not only the nursing staff, but the patients also, would benefit by it. Instruction in bandaging, bed-making, and sick-cookery, will be given to "pupil-probationers" at Tredegar House, and lectures on elementary physiology, anatomy, and hygiene will be given by members of the hospital medical staff. A month's trial in the hospital itself will follow a six' weeks preliminary course before anyone can be enrolled as a "regular probationer" for three years hospital training. This educational scheme will be enforced in the case of all regular probationers as soon as ever the excellent house which has been secured is in order. Hitherto the end of the trial month has been the date from which the period of training was counted, and the salary commenced. Under the new scheme, pupils who are accepted will be reckoned as regular probationers from the actual day of their admission to the wards.

### IN CHARGE OF AN INMATE.

The inquiry into the case of the young man who was seriously burnt on January 28th in Weobley Workhouse will be continued at the next meeting of the Guardians. The evidence of the doctor, as reported in the local journal, showed that the patient was suffering from an attack of chicken-pox, and was ordered to the infectious ward, the doctor directing that someone should be with him, as he was subject to fits. The inmate sent to look after him was a man "in a state of semi-idiocy." The patient was seen on one occasion walking across the yard with no covering save a blanket, and on the day of the accident he was found by another inmate alone, burnt, on the floor. The nurse was in the habit of going to the ward three or four times a day, and both she and the matron consider the semi-idiotic pauper had little influence with his charge.

### GOOD WORK IN SUNDERLAND.

The employment of a third district nurse was advocated by more than one speaker at the annual meeting of the Sunderland Nursing Institute. This branch of the work seems to enjoy universal appreciation, and the committee appeal for increased subscriptions to allow of their extending it. It is satisfactory to note a growing desire that the private and district nursing should be kept distinct, so that all surplus earnings of the former should be used solely for their own benefit, and not be merged in the charitable branch, namely, district nursing.

### A MARTYR TO DUTY.

The death of Nurse Crowley at Westport Workhouse last month is spoken of by the Irish papers as being that of "a martyr to duty." In face of the statement that she nursed several patients night and day until she succumbed to the "malignant fever" from which they were suffering, we are inclined to look upon her untimely death as a proof of bad management. Permitting one woman, however willing, to do the work of two, points to faulty administration, and disregard of their responsibility on the part of those whose duty it is to prevent such "self-sacrifice" of useful lives. In private nursing women are not infrequently expected to do with a minimum of rest, and it seems as if in some Unions they were invited to do without any. A Coventry Guardian remarked the other day that "the nurses were willing to get up at any time of the night when necessary," and he would doubtless consider them necessary "martyrs to duty" if such over work eventuated in mortal illness. People have always had a sentimental admiration for martyrs, and an equal loathing for those who piled up the faggots and applied the torch. If nurses are to be considered martyrs to duty, what are we to think of those who so arrange the duty as to cause the martyrdom?

### AMERICAN SUPERINTENDENTS.

The Convention of the Association of Superintendents of Nursing Training Schools, held last month at Boston, was considered by all present to be a most successful meeting, particularly characterised by the kindly spirit which pervaded the whole of the proceedings. A report of some of the papers read will be published by us in due course, as we can spare space for them. Next year the superintendents propose to hold their conference in Philadelphia, when an equally representative gathering of the leaders of nursing is anticipated.

### SHORT ITEMS.

The annual meeting of the Dukinfield Sick Nursing Association was poorly attended, and increased subscriptions are needed to carry on the work during the year. The nurses' services are much appreciated.—The Battle Board of Guardians have decided to engage a trained nurse at £30 per annum from the Workhouse Infirmary Nursing Association, but are content for their portress to undertake such night nursing as may be required.—The Ladies' Visiting Committee at Bridgwater have addressed a letter to the Board suggesting various improvements in the Nursing Department of the Union.—At the annual meeting of the Kingston Nursing Association it was announced that H.R.H. the Duchess of Albany had consented to become a patroness of the society.—At the annual meeting of the Middlesbrough Nursing Association a vote of thanks was passed to Miss Purvis and the nurses. The senior district nurse, Miss M'Cormack, has been appointed Matron of the Floating Hospital on the Tees.—Miss de Pledge is giving a course of lectures on sick nursing at the South-West London College, Putney Hill.—Miss Ethel Mackenzie gave a lecture on food at the temperance meeting held last month at St. Barnabas' Church House, Kensington.—A bed at the Chelsea Hospital for Women, and one at the Convalescent Home, associated with the institution, has been placed at the disposal of the council of the Newspaper Press Fund for the use of wives and daughters of journalists.—Nurse Sands, of the Chester Infirmary, and Miss Mabel Wollaston, of St. Denys' Home, Warminster, sailed for Tientsin on February 28th to join Bishop Scott's mission in China.—The last report issued of the New Hospital for Women, Euston Road, contains no mention of the nursing staff; this is an unusual omission in the present day, when cordial recognition is generally given in hospital reports of the work of the matron and her staff.

### Elementary Anatomy and Surgery for Nurses.

By W. McAdam Eccles, M.B., M.S., F.R.C.S., Lecturer to Nurses, West London Hospital, &c.

### IX.—SPECIAL FRACTURES.

### Fractures of the Skull.

These, as fractures in general, may be simple or compound. They may involve the vault or base of the skull, or both. The chief anxiety of such injuries lies, however, in the fact that the brain which is enclosed in the skull may, at the same time, be more or less seriously injured. No fracture of the skull should be thought lightly of, and if it be a compound one and chance to become septic, it is a very grave matter indeed.

When a person gets a severe blow or fall on the head, whether the skull be actually fractured or not, he is commonly stunned or concussed. In concussion the whole brain, as it were, is shaken to a greater or less degree, and the resulting symptoms are often very marked. The patient will be found to be quite unconscious or only roused with some difficulty. His face will be pale, his extremities cold, and his pulse small and weak. Often the pupils of the eyes are contracted; if dilated it is a bad sign. A patient may remain in such a state for a considerable length of time; in fact in a most pronounced case he may die without recovering consciousness. But most commonly there will be a gradual return to his normal condition, and one of the earliest signs of the commencement of the favourable turn will often be vomiting, followed later by the reappearance of the patient's colour and warmth. Frequently, he will have no recollection of the accident and its immediate consequences.

A much more serious condition—which may follow a head injury—than mere concussion of the brain is compression of that organ. This in the case of a fracture of the skull is usually brought about by depressed bone, extravasated blood, or inflammatory products. The rapidity of the onset of the symptoms after the infliction of the injury often helps in the diagnosis of the cause of the compression; for, if due to depression of the tables of the skull, they will be immediate; if to hæmorrhage, some length of time—an hour or more —may elapse before they are in evidence, while inflammation takes days to produce them. These symptoms are generally very characteristic. The patient lies on his back, unconscious, "like a log"; all efforts to rouse him are unavailing. He has slow deep snoring breathing, and his pulse is apt to become very infrequent. His face will be more or less livid, and his pupils are generally dilated, and sometimes unequal. If such compression, as evidenced by these symptoms, go unrelieved it will terminate in speedy death; so that compression of the brain may be said to be a surgical emergency.

All patients who have suffered a fracture of the skull need the utmost quiet during their treatment. They should be placed in bed in a cool darkened room, or corner of the ward where they will not be disturbed. Their diet at first must be low, only "slops," and a free action of the bowels should be secured. Shaving off the hair is advised if the temperature rise, and if delirium comes on an ice bag applied to the head is very beneficial. If there happen to be a wound this must be treated strictly antiseptically.

A fracture of the base of the skull is practically always compound. It may usually be recognised by the hæmorrhage which occurs from the ears, nose, mouth, or into the orbits, by the escape of a limpid fluid from the interior of the brain through the same exits, and by the effects of injury to the cranial nerves as evidenced by squints, deafness, &c. A plug of antiseptic wool should be placed very lightly in the ears after they have been syringed out with an antiseptic lotion.

Such fractures very frequently end fatally because of septic infection, inflammation of the membranes of the brain, and of the brain itself being set up. The unconscious state which follows so often on a head injury has to be distinguished from the effects of poison such as alcohol, opium, &c., and also from the results of disease, as diabetes and kidney affection.

### Fractures of the Lower Jaw.

These are fairly common, and usually the result of direct violence. They are always compound, the gum being lacerated. They are very likely to become septic. The moulded splint, if one be used (instead of a dental splint), must be kept accurately adjusted by a four-tailed bandage, and it will be well to see that the patient rinses his mouth out frequently with a weak antiseptic solution.

### Fractures of the Ribs.

An ordinary fracture of one or two ribs may be but a slight affair, and merely calls for treatment by strips of strapping plaster two inches wide and long enough to reach from the spine behind to the sternum in front, applied parallel with the ribs, and when the chest is comparatively empty of air. They should extend below and above the seat of the fracture, and should be supplemented by a broad roller bandage drawn tightly around the chest. This will act by keeping the parts as far as possible at rest. Fractured ribs are united sufficiently firmly in about three weeks.

But a fracture of these bones, especially if the result of direct violence, is very likely to be complicated by injury to the important organs which are situated in the thorax in close contact with the ribs. The lung is most frequently damaged. Evidence of this is soon manifest by the hæmorrhage which occurs. Blood will pass into the air passages themselves, and the patient usually sooner or later coughs up blood. This is termed hæmoptysis, and, if excessive, may lead to death. Again, the blood from the wounded lung may pour out into the chest-cavity, leading to a condition known as hæmothorax. Not only does blood escape from the lung, but air may also pass out, if into the pleural cavity it constitutes pneumothorax, if into the subcutaneous tissue it leads to surgical emphysema. In these cases the tissue just beneath the skin may become greatly blown up with air, which gives to the fingers a crackling sensation when pressed upon. The air may thus pass over the whole body except the scalp, ears, palms of the hands, and soles of the feet. It will in time become absorbed, and only in extreme cases is it likely to do any harm. Serious inflammation of the lungs, especially in old patients who are bronchitic, is apt to follow fractured ribs with a wound of the lungs.

### Fractures of the Bones of the Pelvis.

These are important owing to their liability to be complicated with wounds of the organs in the pelvis. In the nursing of such cases of fracture great care is required for the prevention of bed sores. The utmost cleanliness must of course be observed.

### Fractures of the Spine.

These are most serious accidents, and very frequently terminate fatally. The nurse has a very important *rôle* to play in the treatment of such cases. There is nearly always some injury to the spinal cord leading to paralysis and consequent helplessness on the part of the patient. Bed sores are extremely likely to occur, and it is requisite so as to diminish every needless pressure that the patient be placed on a water or air bed, and that the utmost care be taken to keep him scrupulously clean. No cases perhaps tax the nurse's care and skill more than these fractures associated with paralysis.

### The Royal National Pension Fund for Nurses.

### THE REPORT OF THE COUNCIL.

Next week an account will be given in The Hospital of the eighth general meeting of the members of the Royal National Pension Fund for Nurses. For this meeting at the offices, 28, Finsbury Pavement, on March 14th, the following report was prepared by the council.

The council have the pleasure to submit to the members of the Fund their report, with the annexed accounts and balance-sheet, prepared in conformity with the statutory requirements, and made up for the period of twelve months ending December 31st, 1894.

### Pension Branch.

During the twelve months there have been received: 610 proposals (including 13 brought forward from last account) for granting pensions for £10,854 7s. 3d., of which 54 proposals for assuring the sum of £1,009 10s. are still under consideration or were not proceeded with; 563 policies were granted (7 being in substitution for policies previously existing, leaving 556 net) for assuring £67 6s. 2d. of immediate annuities, and £10 088 18s. 6d. of deferred annuities; producing £3,972 7s. 10d. in single payments, and £6,163 10s. in annual premiums; 3,567 policies have been issued from the commencement of the Fund.

### Sickness Branch.

In this department there have been received: 297 proposals (including 11 brought forward from last account) for weekly sick-pay assurance of £193 17s. 6d., of which 69 proposals for assuring weekly sick-pay of £39 7s. 6d. are still under consideration or were not proceeded with; 228 policies were granted, securing £154 10s. weekly sick-pay, producing £256 11s. in annual premiums; 1,084 policies have been issued from the commencement of the Fund.

### Investments.

The investments on December 31st, 1894, amounted to £198,050 19s. 8d., as follows: In British Government securities, £28,470 10s. 3d.; in colonial (crown colonies) stock, £10,000; in railway and other debentures and debenture stocks, £84,543 2s.; ditto shares (preference and guaranteed), £16,583 18s. 10d.; in foreign government securities, £39,715 8s. 7d.; in municipal corporation bonds and stocks, £8,598 9s.—£187,911 8s. 8d.

### Investments of the Junius S. Morgan Benevolent Fund.

In railway and other debentures, £6,894 10s. 6d.; ditto shares (preference and guaranteed), £2,140; in foreign Government securities, £1,105 0s. 6d.—£10,139 11s. Total invested funds, of both Pension and Benevolent Funds, £198,050 19s. 8d. Cash balances, outstanding interest, loans, &c., £4,375 13s. 6d. Total funds, £202,426 13s. 2d.

### Pension Branch.

It is very satisfactory to report that the figures continue to show a steady growth in all branches of the business. On the pension side 610 proposals were dealt with during the year, as against 533 in 1893; and 556 pension policies were issued, as against 497 in 1893.

As regards withdrawals the council are glad to notice that the proportion is less than in former years, and that several former policy-holders, who had surrendered their policies, have during 1894 again availed themselves of the advantages offered by the Fund.

### Sickness Assurance Branch.

In this branch a steady advance is again observed: 297 proposals being dealt with during the year, as against 260 in 1893; and 228 policies issued, as against 216 in the previous year. The amount of sick pay which was disbursed was £739, as compared with £576 in 1893; the number of nurses amongst whom this was distributed being 149, as compared with 129 in 1893.

### The Junius S. Morgan Benevolent Fund.

The work of this valuable Fund is the subject of a separate report, which will be found on another page, and to which the council think it right to direct attention. It is impossible to over-estimate the value of the services which have been so freely given to this Fund by Lady Rothschild and the other ladies forming the "Advisory Committee." To Miss Pritchard special thanks are due for the zealous and self-sacrificing manner in which she has performed the duties of honorary secretary.

### Council and Officers.

The council have sustained a serious loss by the death of their valued colleague, Mr. Clifford Wigram, late Deputy-Governor of the Bank of England, who was one of their original members, and always took the warmest interest in the prosperity of the Fund.

To supply the vacancy in their number, the council have, in accordance with the articles of association, elected Mr. Herbert C. Gibbs, of the firm of Antony Gibbs and Sons, and are glad to announce that Mr. Gibbs has accepted the nomination.

The following five members of the council retire from office, all of whom, being eligible, offer themselves for re-election: Mr. W. H. Burns, Mr. Edward Rawlings, Mr. A. C. de Rothschild, Mr. Thomas Bryant, and Sir W. H. Broadbent, Bart., M.D.

The articles of association provide that not more than eight representatives of the annuitants and policy-holders of the society may be added to the council. The council have therefore nominated the following ladies for election by the annuitants and policy-holders, and have invited the latter to choose one other representative from their number to serve on the Advisory Committee: Miss C. Davidson, Brompton Hospital for Consumption, Brompton; Miss E. Fisher, General Infirmary, Leeds; Miss L. M. Gordon, St. Thomas's Hospital; Miss K. H. Monk, King's College Hospital; Miss F. C. Nott Bower, Guy's Hospital; and Miss E. Vincent, St. Marylebone Infirmary.

The auditor, Mr. Frederick Whinney, of the firm of Messrs. Whinney, Smith, and Whinney, chartered accountants, retires in accordance with the articles of association, and, being eligible, offers himself for re-election.

### Change of Offices.

The council have, for a long time past, found the offices in King Street inadequate for the growing business of the Fund. It was therefore determined to seek other quarters, and, after considerable inquiry, more commodious premises were secured at 28, Finsbury Pavement, which it is hoped will be found equally central and convenient for the nurses.

During the year the Fund has lost the services of Mr. Pocock, the assistant secretary, who had always been a valuable servant of the institution.

It is satisfactory to be able to report, notwithstanding the large increase in the business of the Fund, that the expenses of management again compare favourably with those of previous years. The council have the satisfaction of testifying to the efficiency of the secretary, Mr. Louis H. M. Dick, and his assistants, who have devoted themselves to the work with commendable energy and success.

By order of the council,

Louis H. M. Dick, Secretary.

The Report of the Junius S. Morgan Benevolent Fund will appear next week.

### Notes and Queries.

The contents of the Editor's Letter-box have now reached such unwieldy proportions that it has become necessary to establish a hard and fast rule regarding Answers to Correspondents. In future, all questions requiring replies will continue to be answered in this column without any fee. If an answer is required by letter, a fee of half-a-crown must be enclosed with the note containing the enquiry. We are always pleased to help our numerous correspondents to the fullest extent, and we can trust them to sympathise in the overwhelming amount of writing which makes the new rules a necessity. Every communication must be accompanied by the writer's name and address, otherwise it will receive no attention.

### Queries.

(95) *Incurable.*—Will you recommend a home where a paralysed lady advanced in years would be received gratuitously?—*Nurse Harrison.*

(96) *Applicant.*—Would a hospital object to receive for training a young Wesleyan who has been a house-parlourmaid?—*A Subscriber.*

### Answers.

(95) *Incurable* (*Nurse Harrison*).—You should write to the Lady Superintendent of St. Luke's House. Osnaburgh Street, London, N.W.

(96) *Applicant* (*A Su scriber*).—Not if she is suited for a nurse's po ition and duties.

### Wants and Workers.

[The attention of correspondents is directed to the fact that "Helps in Sickness and to Health" (Scientific Press, 428, Strand) will enable them promptly to find the most suitable accommodation for difficult or special cases.]

Where can I find a home for two little boys, ages three and six years? Mother dead; father offers to pay 2s. 6d. for each child a week.—*Nurse Harrison.*

### Our American Letter.

(BY A CONSTANT CONTRIBUTOR.)

OTHER matters of nursing interest appear this month to sink into secondary importance, leaving the first place to be rightfully occupied by a report of the Superintendents' Second Annual Convention. This was held in Boston on February 13th and 14th, four sessions taking place; the society may therefore be now considered as in well-established working order.

Its formal title (which is descriptive if somewhat drawn out!) stands as, "The American Society of Superintendents of Training Schools for Nurses." A preliminary meeting of the council was held on Tuesday evening, February 12th, at Boston City Hospital, and the officers for the Convention consisted of the president, Miss Linda Richards; vice-president, Miss Irene Sutliffe; secretary, Miss Mary S. Littlefield; treasurer, Miss Lucy L. Drown; auditors, Miss Diana C. Kimber and Miss Ida Sutliffe; councillors, Mrs. Hunter Robb, Miss Mary A. Snively, Miss M. I. Merritt, Miss Anna C. Maxwell, Miss Gertrude Livingston, and Miss Lavinia L. Dock; committee of information bureau, Miss Emma L. Stowe, Miss Alice Griswold, and Miss McDowell.

At the first meeting, which commenced at 10 a.m. on 13th, the president gave an address, the minutes of the first annual meeting were read, reports received, and other general business transacted. In the course of her address, Miss Richards spoke from her own experience gathered during the twenty years which had passed since she first took charge of a nurse training school in Boston. Schemes for the instruction of nurses were then, Miss Richards remarked, looked upon with suspicion as experimental work, and, by the doctors especially, little hope was entertained of their success. "The change in feeling came slowly, but it has been thorough, and to-day no doctor feels like recalling his old-time prejudices with regard to trained nurses and training schools." Miss Richards' address was most enthusiastically received, and we regret that in the space of a letter we cannot report it at length. Doubtless room will be found for it in THE HOSPITAL later on, when it will not fail to interest many nurses.

In the course of the two hours devoted to the afternoon session on the first day, a paper on the "Uniform Curriculum of Study for Training Schools" by Miss Snively was followed by a discussion led by Miss Darche, in which the suggestions of Miss Snively were considerably criticised. Afterwards an exceedingly comprehensive paper on "Three Years' Course of Training in Connection with Eight Hour System" was read by Mrs. Hunter Robb, late superintendent John Hopkins Training School. A discussion followed, in which Miss Davis, of the University Hospital, Philadelphia; Miss Walker, Presbyterian Hospital, Philadelphia; and others, took part. Eventually a committee was appointed to consider Miss Snively's "Basis of Uniformity" with a view to reporting at the Convention to be held at Philadelphia next year on February 12 and 13. Five members were chosen for this committee, namely: Miss Darche, Miss Brown, Miss Merritt, Miss Snively, and Miss Sutliffe.

In the evening a most enjoyable reception was held by the trustees of the Boston City Hospital.

On the second morning of the Convention the election of officers for next year's meeting took place, and a resolution of regret was afterwards moved by Miss Kimber regarding the death of Miss Perkins, superintendent of the Bellevue Training School, N.Y. Miss Brown, superintendent of nurses at Boston City Hospital, was then called upon to read a paper on "A Plan of Informing Training School Superintendents of Dismissed Pupil Nurses and Probationers." This paper was received with marked approval and Miss Drown, Miss Sutliffe and Miss Davis were nominated as a committee "to formulate a scheme by means of which the plan of information devised by Miss Drown could be supplied to all superintendents."

An interesting paper on "Training School Alumnæ Societies" was contributed by Miss Palmer.

At the afternoon session Miss Dock's paper on "Nurses' Directories" gave rise to a most animated discussion, and it was generally agreed that school registers were the best methods at present for protecting the graduates. Yet it was conceded that a properly-managed central directory might be useful in large cities. It was finally resolved that a committe should represent to the managers of the Belle Vue Training School, the feeling of the Convention "and ask their opinion and co-operation to establish some such scheme in New York, should they deem it possible or desirable."

Miss Littlefield next read a paper by Miss Brennan, who was prevented from attending the Convention, on "Comparative Value of Theory and Practice in Training Nurses."

It was proposed by Mrs. Robb, and seconded by Miss Richards and Miss Darche, that Miss Florence Nightingale should be elected an honorary member of the American Society of Superintendents. A similar compliment being paid to Mrs. Osborne, who had done much at the Belle Vue Hospital and elsewhere, to advance the cause of trained nursing.

In reply to a question from Miss Darche, the President explained that the absence of the paper on "National Organization" was due to the refusals of four matrons who had successively been asked by the Council to write on the subject.

The Convention was concluded by hearty votes of thanks to those who had extended hospitality and courtesy to the superintendents on this memorable visit to Boston. The members present were: Miss Betts, superintendent of nurses, Hahnemann Hospital, N.Y.; Miss Brent, superintendent of nurses, Grace Hospital, Toronto, Canada; Miss Brown, superintendent of nurses, Massachusetts General Hospital, Boston; Miss Darche, superintendent of nurses, Blackwell's Island, New York; Miss Davis, superintendent University Hospital and Training School, Philadelphia; Miss Dock, superintendent of nurses, Illinois Training College, Chicago, Ill.; Miss Draper, superintendent of nurses, Royal Victoria Hospital, Montreal, Canada; Miss Drown, superintendent of nurses, Boston City Hospital, Boston; Miss Elliott, assistant superintendent of nurses, Boston City Hospital, Boston; Miss Griswold, superintendent of nurses, Massachusetts Homœopathic Hospital, Boston; Miss Cross, late superintendent of nurses, Buffalo General Hospital, Buffalo, N.Y.; Miss Hintze, superintendent of nurses, Orange Memorial Training School, Orange, N.J.; Miss Kimber, assistant superintendent of nurses, Blackwell's Island, N.Y.; Miss Littlefield, superintendent of nurses, Episcopal Hospital, Philadelphia; Miss Loomis, superintendent of nurses, Williamsport Hospital, Williamsport, Pa.; Miss McDonell, superintendent of nurses, Elizabeth Hospital, Elizabeth, N.J.; Miss Maxwell, superintendent of nurses, Presbyterian Hospital, N.Y.; Miss McDowell, superintendent of nurses, Newton Hospital, Newton, Mass.; Miss McKechnie, superintendent of nurses, Wilkesbarre City Hospital, Wilkesbarre, Pa; Miss McIsaac, assistant superintendent of nurses, Illinois Training School, Chicago, Ill.; Miss Merritt, superintendent of nurses, Brooklyn City Hospital, Brooklyn; Miss Moore, superintendent of nurses, Lady Stanley Institute, Ottawa, Canada; Miss Nutting, superintendent of nurses, Johns Hopkins Hospital, Baltimore, Md.; Miss Orr, superintendent of nurses,

Paterson General Hospital, Paterson, N.J.; Miss Palmer, late superintendent of nurses, Garfield Memorial Hospital, Washington, D.C.; Miss Plumer, superintendent of nurses, Hartford Hospital, Hartford, Conn.; Miss Quintard, superintendent of nurses, New Haven Hospital, New Haven, Conn.; Miss Richards, superintendent of nurses, Brooklyn Homœopathic Hospital, Brooklyn; Miss Roberts, superintendent of nurses, Syracuse City Hospital, Syracuse, N.Y.; Mrs. Hunter Robb, late superintendent of nurses, Johns Hopkins Training School, Baltimore, Md.; Miss Sanborn, superintendent of nurses, St. Vincent's Hospital, New York; Miss Smith, superintendent of nurses, Maine General Hospital, Portland, Maine; Miss Sniveley, superintendent of nurses, Toronto General Hospital, Toronto, Canada; Miss Stowe, superintendent of nurses, Rhode Island Hospital, Providence, R.I.; Miss I. H. Sutliffe, superintendent of nurses, New York Hospital, New York; Miss I. Sutliffe, snperintendent of nurses, Long Island Hospital, Brooklyn; Miss Walker, superintendent of Nurses, Presbyterian Hospital, Philadelphia.

### Lewisham Infirmary.

In reply to the resolution of the Lewisham Board of Guardians, calling upon the assistant matrons to resign their appointments, these ladies have addressed the following letter to the Infirmary Committee: "In reply to the personal communication received at the Board meeting on February 18th we beg to say that we have no wish to resign our appointments as assistant matrons here. After consideration we must allow having made a mistake in writing as we did to the Local Government Board, seeing that the guardians have regarded the matter as an act of insubordination, of which we should regret to have been guilty. We are and have been in our present position and under trying circumstances quite willing to work under the Local Government Board and the Board of Guardians, and trust that our mode of service in the past will fully bear this out." This letter being considered by the committee "evasive" the assistant matrons were again communicated with, and they wrote in reply as follows: "We much regret that our letter of explanation was not accepted by the Infirmary Committee on Friday, the 1st inst. In deference to your wishes, therefore, we beg to withdraw as far as the Board is concerned the words 'opposition' and 'animosity.' We can only repeat that had we known at the time we wrote the letter to the Local Government Board that it was an act of insubordination we should not have done so. Our ignorance of Poor Law regulations must be an excuse for our action in this matter, and we trust that the Board of Guardians will accept this letter as an evidence of our good faith."

The second letter does not appear to have pleased the committee any more than the first, being again condemned as "evasive," Mr. Wilkinson explaining, according to the local press, that "he did not look upon their communications as constituting an apology such as they would expect from servants who had committed an act of insubordination." At this stage of the proceedings Dr. Pringle is reported to have said that "he had letters in his possession which might affect the whole of their action both with regard to the suspension of the Matron (Miss Patteson) and the assistant matrons." Dr. Pringle's motion that the matter should be referred back to the committee was seconded by Mr. Brunning, who added that "if the whole matter had been thoroughly threshed and investigated the action would not have been taken with regard to the matron and assistant matrons." A report in respect to Miss Patteson's reinstatement had been prepared by the committee, but "was not read in face of Dr. Pringle's warning note."

### Royal British Nurses' Association.

"The pages of the *Nurses' Journal* alone will, in future, bear the official records and announcements of the Royal British Nurses' Association" is the authoritative notice given in the columns of the last issue of the Association's journal. This same number contains Mr. Dent's paper on "The History of Nursing at St. George's Hospital," and a report of the general council meeting of the Royal British Nurses' Association on January 11th.

The Financial Position.

was explained at the meeting, the treasurer reporting that the appeal sent out to each member of the General Council had resulted in an addition to the funds of the Association of £160 a-year for three years. The profits of the bazaar, held in December, under the patronage of H.R.H. Princess Christian, had also substantially improved the financial position of the association.

The Matrons' Council.

In reply to a question as to whether the Matrons' Council was connected with the Royal British Nurses' Association and "the precise position in which the two stood to each other," Mrs. Bedford Fenwick stated "that the Royal British Nurses' Association and the Matrons' Council were entirely independent, although the ultimate object of both was the same, viz., 'The State Registration of Trained Nurses" and ". . . should not be in any way antagonistic, as the matrons who founded the Matrons' Council founded also the Royal British Nurses' Association and the latter stood to the former in the same relation as the General Medical Council to an ordinary Medical Society."

### Hints for Home Nursing.

"We have not a single nurse at liberty" has formed the discouraging answer to many anxious seekers after private nurses during the last few weeks. In those households where trained nursing has not been available the memorandum from the Local Government Board, prepared by Dr. Thorne Thorne, will be particularly welcome. After a summary of influenza epidemics and of the study made of the disease by the Medical Department, the memorandum proceeds to give practical advice as follows:—

"The disease calls primarily for measures of isolation and of disinfection, but there are difficulties in making any such measures universally applicable. Wherever they can be carried out, the following precautions should, however, be adopted:—1st. The sick should be separated from the healthy. This is especially important in the case of first attacks in a locality or a household. 2nd. The sputa of the sick should, especially in the acute stage of the disease, be received into vessels containing disinfectants. Infected articles and rooms should be cleansed and disinfected. 3rd. When influenza threatens, unnecessary assemblages of persons should be avoided. 4th. Buildings and rooms in which many people necessarily congregate should be efficiently aërated and cleansed during the intervals of occupation.

"It should be borne in mind that the liability to contract influenza, and also the danger of an attack, if contracted, are increased by depressing conditions, such as exposure to cold, and to fatigue whether mental or physical. Attention should hence be paid at epidemic periods to all measures tending to the maintenance of health, such as the use of clothing of suitable warmth, and a sufficiency of wholesome food. Persons who are attacked by influenza should at once seek rest, warmth, and medical treatment, and they should bear in mind that the risk of relapse, with dangerous complications, constitutes a chief danger of the disease."

### Death in Our Ranks.

Many old friends of Guy's Hospital will hear with regret of the death of Miss Loaz, who was for 35 years matron of the hospital. She did good work in her own department, and the governors granted her a handsome pension on her retirement in 1879.

### Dress and Uniforms.

By a Matron and Superintendent of Nurses.

### THE LONDON HOSPITAL.

The visitor to the London Hospital cannot fail to carry away, with other agreeable impressions, one of very strong admiration for the neat and becoming uniform worn by the nursing staff. In the centre of the charmingly arranged group of which we give an illustration is seated a "sister," on one side of whom stands a staff nurse, and on the other a probationer. The sister's dress is dark navy blue merino, with a tight-fitting bodice and plainly made skirt, gathered into a band at the waist. Over this is worn an apron of very fine linen, the bibs of which fits close up to the throat, and at the back becomes elongated into straps. These, diverging ingeniously, cross one another, and are kept in position by a tape, on to which they are sewn. This is passed round the waist and tied under the apron in front, hence no possibility of the straps slipping out of the straight line. The cuffs are simply bands of fine cambric with a hem-stitched border top and bottom, and are fastened to the sleeve beneath. A linen collar of the Eton shape is worn with them. The cap fits close to the head, and is trimmed with a double row of gophered lace frilling, and finished off at the back with flowing streamers, edged with the same. The staff nurse wears a very narrow striped lilac print, made plainly, the apron and collar being of the same shape as those used by the sisters. Cambric sleeves reaching to the elbows are worn instead of cuffs, and are pretty as well as useful. An elastic keeps them up, and at the wrist the material is gathered into a narrow band, which fastens with a small pearl button. The cap is a small "mob" shape, trimmed with a double row of gophered fancy Coventry frilling. The probationers garb is quite distinct. Their dress is a small checked lilac print, and an apron with short bib and straps crossing at the back. The pocket of this apron is a particularly useful one; it is shaped somewhat like a pouch, and is inserted into the waistband, so that there is no chance of its tearing or catching in things, as pockets which are worn at the sides have an unfortunate habit of doing sometimes. The cap is similar in shape to that of the staff nurses, and it is trimmed with two rows of plain Coventry frilling, the top row of which stands up, the lower falling in the contrary direction. Messrs. Debenham and Freebody, who supply the hospital with its nurses' uniforms, are to be congratulated on the finish and style they have shown in bringing to perfection every detail of the costume.

### An Action at Anerley.

In an action for slander recently brought by a nurse against a former employer it was shown that during her engagement the nurse had given satisfaction to patient and doctor. After her recovery the lady had made and written certain statements which were said to constitute the slander. Eventually an amicable settlement was arrived at, the defendant agreeing to pay the nurse £30 and taxed costs. Mr. Justice Wright approved of the settlement, and considered that, as a nurse, Miss Ann Elizabeth Brierley was free from blame, and the defendant's statements, "perhaps made incautiously, were made without malice."

### Spring Novelties.

The dark days of winter are drawing to a close, March warning us that at any moment spring may burst forth in all the warmth and in all the beauty we are accustomed to associate with that delightful season of the year. In order that the first sunshiny days shall not find us altogether unprepared it may be well to consider beforehand the question of lighter clothing. Apart from the discomfort entailed, it is false economy to continue wearing winter garments after they have ceased to be necessary. In the first place they are always a more expensive item than any other in the expenditure; and in the second, a hot sun very quickly makes havoc with what has already done duty for so many months. When the warm weather seems fairly well established it is a good plan to look carefully through the heavier articles of wearing apparel, and after carefully brushing them and sponging with spirits of ammonia those that are black, to fold them up and put them away till the following winter, with one or two pieces of camphor to keep the moth away. It is wonderful how fresh and sweet they turn out when next required. In the meanwhile it will be necessary to look about for something more suitable to replace them with. Messrs. Debenham and Freebody have a very good selection, and among their specialities for nurses are showing a sweet little Princess bonnet, trimmed with velvet, and relieved with a neat white border and tucked strings. They have a large assortment of other novelties as well which amply sustain their deservedly high reputation. A visit to Messrs. E. and R. Garrould of the Edgware Road is one which might be paid with advantage. A large quantity of costumes, mantles, and cloaks, are now on view, and busy people like nurses can very quickly be fitted *cap-à-pied* in the best style.

Harrod's stores are also doing a brisk trade in this special line at present, and as the holiday season comes round again with the approach of Easter, many little requirements suggest themselves as coming in handy bye-and-bye. For those who wish to cast aside uniform while away from their professional duties there are walking dresses, Calder capes, and a variety of homespuns, serges, and Scotch tweeds, which cannot fail to delight the heart and taste of the wearer. Boots and shoes are another consideration, and tender feet are best shod in those so well described as "hygienic." Messrs. J. and R. Dick (Holborn), Messrs. Mansfield and Sons (Strand), and the London Shoe Company (New Bond Street) are all to be congratulated on the perfection of style and comfort attained by this kind of boot which they have done so much to popularise. Fleming Reid and Co., of the Greenock Mills, as usual have an attractive display of woven goods. Natural wool combinations and vests are almost a necessity for workers in an institution where frequent and rapid changes of temperature are inevitable. Their wonderful cheapness, too, is a qualification that will ensure for them a ready sale. A very enterprising firm is that of T. R. Roberts (Upper Street, Islington), and excellent in taste and variety are their ready-made costumes. We can with every confidence recommend to our readers a charming nurse's bonnet in black straw trimmed with velvet, which they have recently brought out at a very low price, and which will be found becoming to most faces.

Few establishments, perhaps, have risen more rapidly to eminence and popularity during the last decade than that of Mr. Peter Jones (Sloane Square). His linens, calicos, and flannels are all of first-rate quality, and the choice is extended over a wide range of articles of equal excellence. If the spacious premises included but a nurses' uniform department, they would be complete, as, from the number of hospitals in the neighbourhood, patronage would be certain. The Royal Serge Warehouse (Egerton Burnett) is showing some excellent material just now, suitable for spring costumes. The special merit of these goods lies in their durability, lightness, and cheapness. They retain their colour and appearance to the end. Those of our readers who are possessed of nimble fingers and are clever at cutting out cannot do better than send for a length and provide themselves, at a small cost with a useful dress.

### Nursing Appliances.

Messrs. Hockin, Wilson, and Co. (186a, Tottenham Court Road) have just brought out a delightful bag, suitable for a district nurse. It is quite small and very light, and contains everything necessary in an emergency. The fittings are very complete, even to the feeder and kidney bowl. The bag is in dull leather with nickel mounts, and is so cheap that there need be no hesitation about its purchase on that score. Many other useful articles would render a visit well worth the trouble, as it is impossible for a description, however graphic, to deal adequately with the matter. Mr. W. K. Stacey, (4, Newgate Street) another firm to whom nurses must feel grateful, have a large selection of wallets and chatelaines on view just now. The prices vary according to the finish and quality of the article, and the fittings can be selected according to the requirements of the purchaser, an advantage which is at once obvious. For travelling trunks and dressing bags, few can compete with Mr. S. Fisher (Strand), whose reputation for these articles stands deservedly high. A catalogue will be forwarded post free on application to any address, giving full details as to size, fittings, and cost. Allen and Hanbury's pocket case for nurses is one of the most convenient articles of the kind that has hitherto been issued. It folds into a small compass and can be easily carried in the pocket. Their chart book for the registration of temperature, &c., is a requisite which no private nurse should be without. The illustrated catalogue issued by W. H. Bailey and Sons contains a very comprehensive description of instruments and wallets for nurses. The "dust proof" wallet is a most ingenious contrivance and well worthy of patronage. Their district bags are also to be commended as replete with all that the heart of a nurse can desire.

### A New Soap Tray.

Matrons and others concerned in household matters will welcome the neat little contrivance invented by Messrs. Norris for attachment to pails, washing tubs, or other articles in conjunction with which soap is used. The waste which accrues, besides the unnecessary diving into unclean water when the soap is allowed to remain in the pail, is an obvious objection, but a plan which is followed by only too many persons; The "Little Friend Soap Tray" will remove the necessity for any such course, at the expenditure of but a few pence. The tray, made in zinc, with two simple hooks to hang on to the side of the pail, can be had, perforated or not, at the purchaser's desire. It is large enough to hold the flannel also; and doubtless any modifications which suggest themselves to the practical would be willingly adopted by manufacturers so evidently alive to everyday needs of the worker. The makers are Messrs. Norris and Sons, 5, New Street, Bishopsgate, E.C.

### The Nurse and the Sister.

### I.—THE NURSE'S STANDPOINT.

Who's after me from morn till night
To see if I am doing right,
And seems possessed of second sight?
My Sister.

Who knows by instinct when I'm late,
Five minutes past the stroke of eight,
And for excuses will not wait?
My Sister.

Who plans my duties one by one,
Nor lets me go until they're done,
And sees that I've forgotten none?
My Sister.

Who scans the ward with critic's eye
To see if anything's awry,
Includes me in her scrutiny?
My Sister.

Who looks me o'er with glance most keen,
And thinks my apron not too clean,
And wonders where my cap has been?
My Sister.

Who thinks I do not know my work,
And fancies a desire to "shirk"
Within my breast is sure to lurk?
My Sister.

Who's always sure to be about
Just when the spirit lamp is out,
Sees the steam kettle's steamless spout?
My Sister.

Who finds a medicine's overdue,
And makes me poultices renew
That were "all hot" an hour ago?
My Sister.

Who says the fomentation's not
Put on just right, nor boiling hot,
Nor yet on the affected spot?
My Sister.

Who thinks I should no temper show,
No matter what she likes to do,
And not say "Yes" if she says "No"?
My Sister.

Who scolds me if some dust she find
On tables, books, or window-blind.
And tells me I "had better mind"?
My Sister.

Who's sure do see the sheet that's torn,
The shirt that's of its buttons shorn,
And makes me mend whatever's worn?
My Sister.

But who perceives with equal ease
Each effort that I make to please,
And every small improvement sees?
My Sister.

Who due allowances will make
For tired feet or bad headache,
Causing omission or mistake?
My Sister.

Who's always kind and ever just,
And only scolds because she must;
Is good to those whom she can trust?
My Sister.

And who, when there is trouble near,
Will prove herself a friend most dear,
Helping me on with words of cheer?
My Sister.

To whom then should I loyal be,
Condone the faults that I may see,
For sake of all she is to me?
My Sister.

### II.—THE SISTER'S STANDPOINT.

Who puts her feelings into verse,
In rhyming phrases short and terse,
That could not be a great deal worse?
My Nurse.

Who's given me a chance to say,
The life *she* leads me day by day,
While I'm on duty's toilsome way?
My Nurse.

Who misconstrues my ev'ry act,
Who thinks me horribly exact,
Tyrannically hard, in fact?
My Nurse.

Who seems to think that she knows best,
And yet, when put to sudden test,
Does nothing better than the rest?
My Nurse.

Who's often late, day after day,
And yet if but one word I say
Looks gloomily the other way?
My Nurse.

Who thinks I'm ever on the prowl
For opportunities to growl,
And greets me with portentous scowl?
My Nurse.

Who breaks the vases that I buy,
The flower-pots that I, too, supply,
Who sometimes works most carelessly?
My Nurse.

Who wants some "special leave," of course
Just when a patient's taken worse?
Who my decisions would reverse?
My Nurse.

Who cares not for "self-sacrifice,"
But loves to do the thing that's nice,
And wants "free time" at any price?
My Nurse.

Who's irrepressible at times,
Commits all manner of small crimes,
Would laugh if she could see these rhymes?
My Nurse.

But who, if I am ill or tired,
Is everything to be desired,
And with devotion's quickly fired?
My Nurse.

Who makes me arrowroot at night,
And sees that everything's just right,
And does her work with all her might?
My Nurse.

Who's loyal to me though I scold
Her often, if the truth be told,
Who on my heart has got a hold?
Ny Nurse.

Who is it that I don't report
As often, as I fear I ought,
Because forgiveness she has sought?
My Nurse.

Who really loves me, then, although
I may not much affection show?
Who's sure I am her friend, I know?
My Nurse.

### Everybody's Opinion.

[Correspondence on all subjects is invited, but we cannot in any way be responsible for the opinions expressed by our correspondents. No communications can be entertained if the name and address of the correspondent is not given, or unless one side of the paper only be written on.]

### SNAKE-BITE IN AUSTRALIA.

A correspondent writes:--Professor Halford, M.D., of the Melbourne University, who for thirty years has been investigating the history and cure of snake-bite, and is the author of the so-called ammonia cure, now recommends chloride of lime; the powder is diluted in twelve parts of water, and injected by means of the hypodermic syringe. Several cases of cure are already reported from country towns. In view of the fact that the injection would be a dangerous operation in inexperienced hands, Dr. Gresswell, Chairman of the Central Board of Health, has declined to issue instructions for its employment. He rather advises that, whenever possible, the services of a duly qualified medical practitioner should be obtained. It is to be observed, however, that liability to snake-bite is most frequent in those solitary parts of the country where medical assistance is most difficult to obtain. The question of snake-bite and its cure is likely to remain a pressing one to Australians for several generations yet, in fact, until the country is thickly settled and reptiles exterminated.

### PRIVATE NURSING.

L. W. R. writes: A letter from a nurse appeared in The Hospital, February 16th, complaining that she has been out of employ for fourteen weeks, and that she knows many nurses in similar difficulty. She gives as a reason that 5,000 ladies of independent means are occupying paid positions to the exclusion of others wl o need employment. May I suggest that there are other reasons why some nurses do not find work? A week or two since, requiring a nurse-attendant for an invalid, I answered several advertisements in The Hospital. Some nurses did not reply, others called, and of these, two entirely declined to dust and keep the patient's bedroom in order, two refused to take their meals with the maids, and all said they would do nothing menial. In fact, only one was willing to undertake the entire charge of the invalid and his room, although £50 a year and laundress was offeied.

### Appointments.

City of London Lying-in Hospital.—Miss Annie Fox, who was trained at St. Mary's Hospital, where she afterwards held the post of Sister, has been appointed Matron of the City of London Lying-in Hospital. We congratulate Miss Fox on her promotion.

Bromley Cottage Hospital, Kent.—Miss Bessie Coleridge Davis has been made Matron of this hospital. She was trained at King's College Hospital, where she afterwards held the post of night sister. Miss Davis has our best wishes for success in her new work.

Charnwood Forest Convalescent Home.—Miss Georgina Napier has been appointed matron of this Convalescent Home. She was trained at the Royal Hants County Hospital, Winchester, and held the post of ward sister there for six months. Miss Napier has taken holiday duty in the Royal South Hants Infirmary, the London Temperance Hospital, and Wolverhampton Eye Infirmary, and for one year held the position of ward sister at Derby Royal Infirmary. We congratulate her on her appointment.

### MEDICO-PSYCHOLOGICAL ASSOCIATION.

The next examination will be held on Monday, May 6th, 1895. Schedules, to be filled up by the candidates, can be obtained from the Registrar, Dr. Spence, Burntwood Asylum, Lichfield, and must be returned to him at least four weeks before the examination.

### Nursing in Holland.

At Rotterdam a "Society for Rendering First Aid in Cases of Accident" has been established and already numbers some two hundred members; the committee consists of three medical and three lay members, and the president, Dr. M. Polak.

Under the presidency of Deaconess De Gaay Fortman the second general meeting of the Reformed Nursing Union was held, a short time ago, in the Nieuwe Kerk, Amsterdam. The report presented to the meeting by the secretary was a decidedly favourable one, the number of members having risen to two hundred, while the income of the year exceeded the ordinary expenditure by the sum of 800 francs. The president announced to the meeting that in order to cover the expense necessarily incurred in the purchase of a house, it was proposed by the committee to raise a loan, and after some discussion this proposal was unanimously agreed to.

On February 1st a new institution for the care of the mentally afflicted was opened at Scheveningen, in the Villa Mignon. The head of this establishment, Herr A. F. Henken, is a certificated mental nurse, and has had long experience in the asylum at the Hague.

A hospital has lately been opened at Beetsterzwaag, which, although on a small scale, seems to be doing excellent work. The very poor are received free; those who can afford it, however, pay a small sum. Persons who enter the hospital entirely destitute are, on their recovery, provided with clothes.

### Where to Go.

"Wandering Minstrels" will give a concert at the Star and Garter, Richmond, on Thursday, March 21st, for the benefit of the building fund of the Princess May Ward for Children, Royal Hospital, Richmond. Their Royal Highnesses the Duke and Duchess of Teck have promised to be present at the entertainment.

Windsor Castle.—On and after Monday, March 18th, the State Apartments will be open to the public on weekdays, Wednesdays excepted, until further orders.

Sir Dyce Duckworth will lecture on "The Modern Trained Nurse," at eight p.m., on Friday, March 15th, at 17, Old Cavendish Street.

The Amateur Art Exhibition will take place in May in aid of the Parochial Mission-Women Fund, the East London Nursing Society, and the East End Mothers' Home. Those who intend to exhibit should communicate with the Hon. Secretary, the Hon. Mrs. Charles Eliot, 8, Onslow Gardens.

St. Anne's Church, Soho.—Bach's "Passion" (St. John) on Fridays during Lent at eight p.m. Doors open at half-past seven. Admission free, by tickets obtained from the rectory. Liberal offerings are asked. If, after paying the heavy expenses, there is any surplus it is given to the poor fund.

East-end Mothers' Home.—Annual meeting at Lowther Lodge, Kensington Gore, Monday, March 18th, at three p.m.

Guildhall.—Saturday afternoon, 16th inst. At a concert in aid of the widows and orphans of fishermen lost in the recent gales, the following artistes will be amongst the performers: Messrs. Sims Reeves, Iver McKay, F. Davies, Johannes Wolff, and Charles Fry, Mesdames Palliser, Sterling, and the Meister Glee Singers.

Paddington Green Children's Hospital.—A series of entertainments and a bazaar will be held at this hospital on March 28th, 29th, and 30th. The Right Hon. the Countess of Warwick will open the bazaar at half-past two p.m. on the first day, and the Countess of Harrowby on the second.

### An Acceptable Gift.

Through the kindness of Mrs. Elkington and other friends the nursing staff of the Ipswich and East Suffolk Hospital have been provided with a handsome new piano, a duet stool, and other useful articles for the nurses' room.

## Figures and Tables

**Figure f1:**
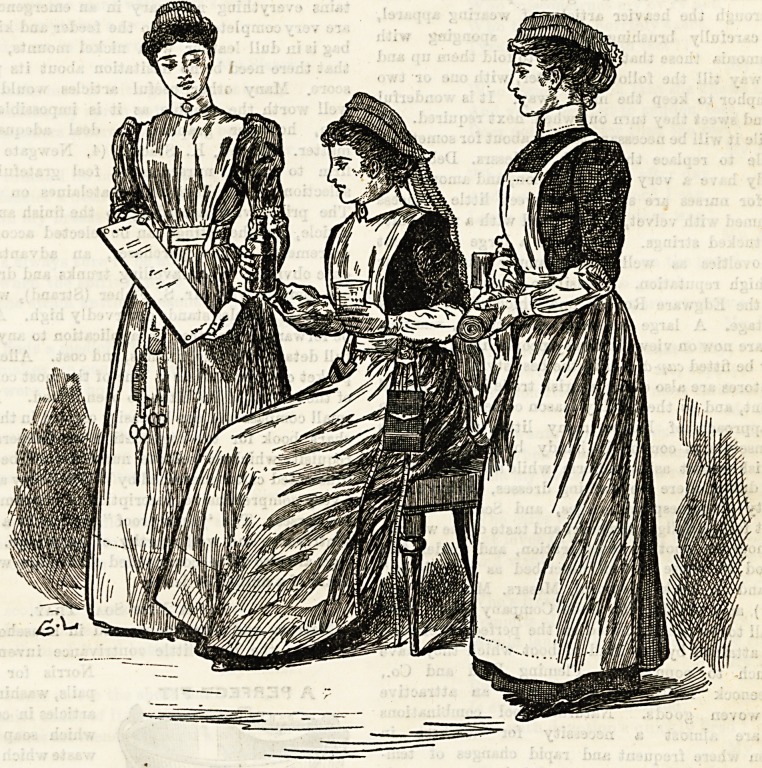


**Figure f2:**